# Managing Acute Portal Hypertensive Gastropathy Bleed During the Time of COVID-19 Pandemic: Novelty or Necessity?

**DOI:** 10.7759/cureus.8333

**Published:** 2020-05-28

**Authors:** Cyriac A Philips, Sandeep Kumbar, Rizwan Ahamed, Philip Augustine

**Affiliations:** 1 Gastroenterology, Cochin Gastroenterology Group, Kochi, IND; 2 Gastroenterology, Ernakulam Medical Center, Kochi, IND

**Keywords:** gi bleeding, covid, coronavirus, endoscopy, evl, banding, cirrhosis, phg

## Abstract

Acute bleeding from portal hypertensive gastropathy (PHG) is an extremely rare event in the natural history of cirrhosis. The treatment recommendations include portal pressure reduction strategies including pharmacotherapy with vasoactive agents and beta-blockers and interventional strategies such as transjugular intrahepatic portosystemic shunt placement. In this report, we present the case of a patient with cirrhosis in whom acute PHG-related bleed was managed with endoscopic band ligation, a therapeutic modality which has not been described in current literature. Our decision to re-purpose a technique for variceal bleeding stems from the fact that during the ongoing COVID-19 pandemic, the technical assistance, resource availability, and sourcing of materials that were required for us to follow recommended management guidelines for acute PHG-related bleed was severely affected due to imposed lockdown between districts and states.

## Introduction

Portal hypertensive gastropathy (PHG) occurs in cirrhosis patients with advanced liver disease and its occurrence and symptomatic presentation escalate with the severity of underlying portal hypertension. It is more likely to be seen in cirrhosis patients with esophageal varices and in those with a history of endotherapy for esophageal varices. The commonest presentation is that of an asymptomatic patient identified during an endoscopic evaluation. A close differential is gastric antral vascular ectasia (GAVE) in which the endoscopic appearance is unique and that of red spots arranged in either lines running proximally from the pylorus or as a diffuse nodular pattern with antral predominance and acute bleeding presentation from GAVE is almost nonexistential [1]. In PHG, a significant number of symptomatic patients exhibit features suggestive of chronic gastrointestinal bleeding and iron deficiency anemia similar to GAVE. Nonetheless, less than three percent of PHG patients can present with an acute bleed [2]. At present, there is no clarity in recommendations for emergency treatment of an acutely bleeding PHG patient. In this report, we present the case of an acute PHG bleed managed with targeted endoscopic band ligation of the bleeding gastric mucosa, a never before reported therapeutic consideration in patients with active PHG-related bleed. Our decisions to perform an age-old technique in a novel scenario rose from reasons related to resource and technical scarcity due to the ongoing coronavirus pandemic. 

## Case presentation

**Figure 1:**
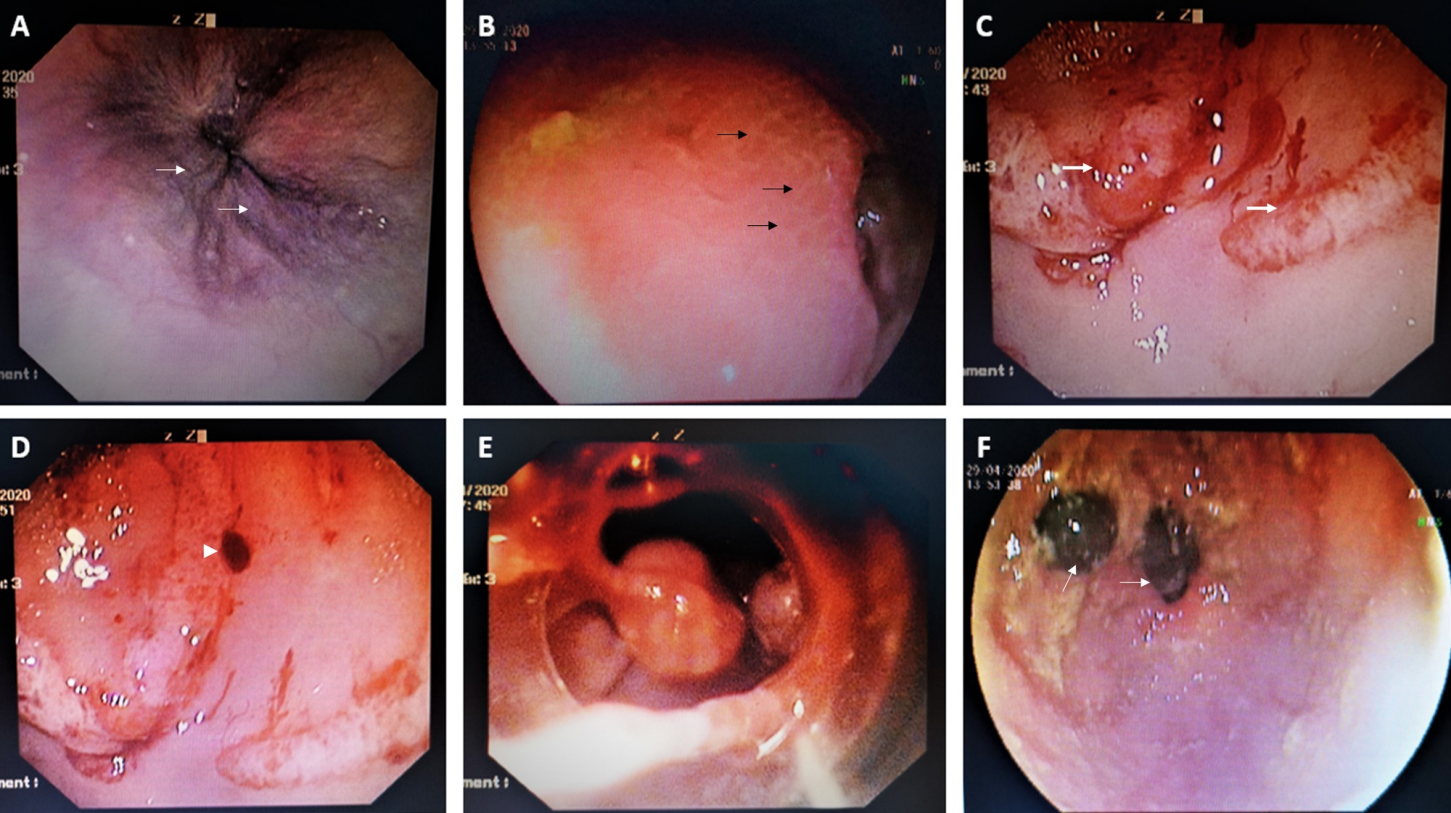
Endoscopic band ligation of actively bleeding portal hypertensive gastropathy. Urgent upper gastrointestinal (GI) endoscopy showed small low risk esophageal varices without stigmata of recent hemorrhage or active bleeding (A, white arrows) while the gastric mucosa showed extensive mosaic patterning (B, black arrows) of the fundus and body and confluent red spots associated with continuous oozing (C, thick white arrows) and difficult to dislodge fresh clots (D, white arrowhead) suggestive of severe acute bleeding portal hypertensive gastropathy (PHG). Endoscopic band ligation of the bleeding PHG mucosa was performed at targeted points (E) leading to complete hemostasis as seen on relook endoscopy 24 hours later (F, white arrows).

A 46-year-old man diagnosed as a case of decompensated alcoholic cirrhosis with prior history of endoscopic band ligation for acute variceal bleeding and with controlled complications of portal hypertension on medical management presented to the gastrointestinal unit with complaints of acute onset episodes of melena with severe lethargy and giddiness for one day. The patient had been abstinent from alcohol for over two years. The patient denied fever, cough, throat pain, abdominal pain, hematemesis, and pain-killer medicine use. His current medications included carvedilol (6.25 mg twice daily), rifaximin (550 mg twice daily), zinc supplements, low dose diuretics (torsemide 5 mg with spironolactone 25 mg four days a week), and oral lactulose. On evaluation, the patient was conscious and oriented, but lethargic, pale, and icteric without asterixis. His blood pressure was 100/54 mmHg with a pulse rate of 70 per minute. Investigation showed hemoglobin 7.8 g/L, thrombocyte count 66 × 109/L, total bilirubin 3.1 mg/dL, direct bilirubin 1.2 mg/dL, and international normalized ratio 1.92 with serum sodium 129 mmol/L. Evaluation for sepsis was noncontributory and with model for end stage liver disease (MELD) score 18 and Child-Pugh score 10. Ultrasound of the abdomen revealed features of cirrhosis and portal hypertension with mild ascites. A review of the recently performed abdominal CT did not reveal ectopic varices, hepatocellular carcinoma, or portal vein thrombosis. Intravenous terlipressin was initiated (2 mg bolus followed by 1 mg every six hours) along with prophylactic intravenous ceftriaxone. Urgent upper gastrointestinal endoscopy revealed small low risk esophageal varices without stigmata of recent hemorrhage or active bleeding (Figure 1A). The gastric mucosa showed extensive mosaic patterning (Figure 1B) of the fundus and body, extending to the antral region with confluent red spots associated with continuous oozing (Figure 1C) and difficult to dislodge fresh clots (Figure 1D), features suggestive of severe acutely bleeding PHG. In view of ongoing melena and need for blood transfusion, salvage treatments were contemplated. Injection sclerotherapy of the bleeding gastric mucosa was not performed in view of advanced liver disease and endoscopic cauterization could not be performed due to the extend of the disease, active bleeding and absence of technical assistance, latter due to intense government-initiated lockdown against COVID-19 within the district. Transjugular-intrahepatic portosystemic shunt (TIPS) placement could not be performed due to the absence of TIPS stents in stock. All inter-state services were halted by the respective state governments due to the COVID-19 emergency and procurement of medical stents were impossible. Need for urgent liver transplantation, in the event of the ongoing severe uncontrolled bleed led to the worsening of decompensation with medical management alone, was discussed with the family but they refused. Hence the decision to perform endoscopic band ligation of the bleeding PHG mucosa was made and using the universal Saeed® multi-band ligator (Cook Medical, Bloomington, USA) the actively bleeding gastric mucosa at targeted points were banded under endoscopic guidance (Figure 1E). A relook endoscopy 24 hours later revealed complete hemostasis and controlled mucosal necrosis at the ligation site (Figure 1F).

The patient was discharged to home the following day.

## Discussion

We present the case of a patient with cirrhosis with actively bleeding PHG. In current literature, the management of PHG-related acute bleeding consists of optimization of beta blockers and the use of terlipressin, octreotide, or somatostatin for portal pressure reduction in line with acute variceal bleed management. In uncontrolled active PHG-related bleeding, endoscopic cauterization and injection sclerotherapy have been advocated by some authors, even though the former has been studied only in chronically bleeding PHG and latter fallen out of practice in advanced liver disease with the advent of TIPS and liver transplant surgery. In extreme cases, TIPS placement, shunt surgery, and liver transplantation have been recommended [3]. There are no studies or reports on the use of band ligation for controlling acute PHG-related bleeding. In our patient, due to circumstantial exhaustion of recommended treatments due to the COVID-19 pandemic, we were forced to decide on a known technique in a new situation which ultimately proved successful. Worsening decompensation and increased need for endoscopic treatments in patients with cirrhosis during fasting have been documented before [4-5]. Our report signifies the need for necessitating and re-purposing the known techniques in well-known, but difficult clinical scenarios during a time, such as the current pandemic, when resources and personnel become unexpectedly restrained. 

## Conclusions

In resource and technical assistance constrained situations, endoscopic band ligation for acute PHG-related bleed may be a safe and novel alternative until definitive management can be undertaken, which requires confirmation in prospective studies.
